# Supplementary material for: Competing scaffolding proteins determine capsid size during mobilization of Staphylococcus aureus pathogenicity islands
Source: eLife. 2017 Oct 6;6:e30822. doi: 10.7554/eLife.30822 (PMC5644958; doi:10.7554/eLife.30822)
Supplement: Supplementary file 1. — Restriction sites are underlined. [file elife-30822-supp1.doc]

| Plasmid | Oligonucleotide | Sequence | Description |
| --- | --- | --- | --- |
| pEW3 | EAW5 | CGATGCATGCCATGGTGGTCGAAAACAAGGACTTTAGCGATAG | pMAD derivative for constructing |
|  | EAW6 | AATAATTTTTTTTTGTCTAGCTATTTCAGCTAAGTTTTGCG | ST196 (80α SP::R202K) |
|  | EAW7 | CAAAAAAAAATTATTAAAAATTAACGGAGGCATTTAAATGGAAC |  |
|  | EAW8 | GGCGATATCGGATCCTTCCAATGATTTCGGGCATGTTAC |  |
| pEW14 | EAW58 | CGATGCATGCCATGGTTAATAGCTAGCACTTAATTGTGTTGGC | pMAD derivative for constructing |
|  | EAW59 | TTAAACTTCTCCTGGTTGTGTTTGTTCCATTTAAATGCCTCC | ST248 (80α Δ*CP)* |
|  | EAW60 | CCAGGAGAAGTTTAATAAACAATTAGGAGTGG |  |
|  | EAW61 | GGCGATATCGGATCCATATCTCAAAAGAACACAGCCCTTCC |  |
| pEW19 | EAW5 | CGATGCATGCCATGGTGGTCGAAAACAAGGACTTTAGCGATAG | pMAD derivative for constructing |
|  | EAW72 | AATAATTGATTTTTGTCTAGCTATTTCAGCTAAGTTTTGCG | ST278 (80α SP::R202S) |
|  | EAW73 | CAAAAATCAATTATTAAAAATTAACGGAGGCATTTAAATGGAACAAAC |  |
|  | EAW8 | GGCGATATCGGATCCTTCCAATGATTTCGGGCATGTTAC |  |
| pEW20 | EAW5 | CGATGCATGCCATGGTGGTCGAAAACAAGGACTTTAGCGATAG | pMAD derivative for constructing |
|  | EAW74 | AATAATTTCTTTTTGTCTAGCTATTTCAGCTAAGTTTTGCG | ST279 (80α SP::R202E) |
|  | EAW75 | CAAAAAGAAATTATTAAAAATTAACGGAGGCATTTAAATGGAACAAAC |  |
|  | EAW8 | GGCGATATCGGATCCTTCCAATGATTTCGGGCATGTTAC |  |
| pLAK1 | EAW5 | CGATGCATGCCATGGTGGTCGAAAACAAGGACTTTAGCGATAG | pMAD derivative for constructing |
|  | EAW159 | TTTAATCGTTCTTTTTTGTCTAGCTATTTCAGCTAAGTTTTGCG | ST358 (80α SP::I203T) |
|  | EAW160 | AAAAGAACGATTAAAAATTAACGGAGGCATTTAAATGGAACAAAC |  |
|  | EAW8 | GGCGATATCGGATCCTTCCAATGATTTCGGGCATGTTAC |  |
| pLKP2 | EAW58 | CGATGCATGCCATGGTTAATAGCTAGCACTTAATTGTGTTGGC | pMAD derivative for constructing |
|  | EAW237 | GTTTTCTAAAACCTCTTGTAAGATGGGCGTTG | ST384 (80α CP::M52L) |
|  | EAW238 | GAGGTTTTAGAAAACTCTAAAATTATGCAATTAGGTAAGTACGAACC |  |
|  | EAW239 | CTCCCGGGTACCATGGATATCTCAAAAGAACACAGCCCTTCC |  |
| pLKP3 | EAW58 | CGATGCATGCCATGGTTAATAGCTAGCACTTAATTGTGTTGGC | pMAD derivative for constructing |
|  | EAW240 | TTGTGAACAAGTATAATTCAAGAATTCTTTTGTTACAGGTAAGATAACC | ST385 (80α CP::Y123C) |
|  | EAW241 | TATACTTGTTCACAATTCTTTGAAGAAATGAAGCCTATG |  |
|  | EAW239 | CTCCCGGGTACCATGGATATCTCAAAAGAACACAGCCCTTCC |  |
| pLKP14 | EAW58 | CGATGCATGCCATGGTTAATAGCTAGCACTTAATTGTGTTGGC | pMAD derivative for constructing |
|  | LKP10 | GTTTTCTTGAACCTCTTGTAAGATGGGCG | ST415 (80α CP::M52Q) |
|  | LKP11 | GAGGTTCAAGAAAACTCTAAAATTATGCAATTAGGTAAGTACGAACC |  |
|  | EAW239 | CTCCCGGGTACCATGGATATCTCAAAAGAACACAGCCCTTCC |  |
| pLKP15 | EAW5 | CGATGCATGCCATGGTGGTCGAAAACAAGGACTTTAGCGATAG | pMAD derivative for constructing |
|  | LKP12 | TTGTCTAATTATTTCAGCTAAGTTTTGCGGTTTATTTTTAGT | ST417 (80α SP::A198I ) |
|  | LKP13 | GAAATAATTAGACAAAAAAGAATTATTAAAAATTAACGGAGGCATTTAAATG |  |
|  | EAW239 | CTCCCGGGTACCATGGATATCTCAAAAGAACACAGCCCTTCC |  |
| pLKP31 | EAW5 | CGATGCATGCCATGGTGGTCGAAAACAAGGACTTTAGCGATAG | pMAD derivative for constructing |
|  | GC121216B | TAGCTAAATTTTGTAGCGTTGTACCATATTGCTTTTGCTTGTGATTAAATGAATCTCCACCAGTCAATGG | ST466 (80α SP::CpmB_CTD_) |
|  | GC121216A | AACGCTACAAAATTTAGCTAAGCAAAACAGGATTATTAAATAG CG GAG GCA TTT AAA TGG AAC AAA CAC |  |
|  | EAW239 | CTCCCGGGTACCATGGATATCTCAAAAGAACACAGCCCTTCC |  |
| pLKP32 | GC121316A | CGATGCATGCCATGGGCTATACAAGAATTGGAACAAGAGCGTAATGC | pMAD derivative for constructing |
|  | GC121316B | GTCTAGCTATTTCAGCTAAGTTTTGCGGTTTATTTTTAGTCGAAGACTGTTCTGATTGTTGTAACTCACTTGAAATC | ST458 (80α) SaPI1 CpmB::SP_CTD_) |
|  | GC121316C | CTTAGCTGAAATAGCTAGACAAAAAAGAATTATTAAAAATTAA CAATGATTGCCTATCCAATTCGGGTAG |  |
|  | GC121316D | CTCCCGGGTACCATGGCTCGTGCAATCTTACTGTTTTCAATTGCTG |  |
