# Supplementary material for: Competing scaffolding proteins determine capsid size during mobilization of Staphylococcus aureus pathogenicity islands
Source: eLife. 2017 Oct 6;6:e30822. doi: 10.7554/eLife.30822 (PMC5644958; doi:10.7554/eLife.30822)
Supplement: Transparent reporting form [file elife-30822-transrepform.docx]

***eLife’s* transparent reporting form**

We encourage authors to provide detailed information *within their submission* to facilitate the interpretation and replication of experiments. Authors can upload supporting documentation to indicate the use of appropriate reporting guidelines for health-related research (see [EQUATOR Network](http://www.equator-network.org/%20)), life science research (see the [BioSharing Information Resource](https://biosharing.org/)), or the [ARRIVE guidelines](http://www.plosbiology.org/article/info:doi/10.1371/journal.pbio.1000412) for reporting work involving animal research. Where applicable, authors should refer to any relevant reporting standards documents in this form.

If you have any questions, please consult our Journal Policies and/or contact us: [editorial@elifesciences.org](mailto:editorial@elifesciences.org).

**Sample-size estimation**

- You should state whether an appropriate sample size was computed when the study was being designed
- You should state the statistical method of sample size computation and any required assumptions
- If no explicit power analysis was used, you should describe how you decided what sample (replicate) size (number) to use

Please outline where this information can be found within the submission (e.g., sections or figure legends), or explain why this information doesn’t apply to your submission:

• The study used 10,000-14,000 particle images, which were classified by standard correlation-based methods using an unbiased starting model

• The Fourier shell correlation between two independent data subsets was used to determine resolution, which is also dependent on the size of the data set.

• The procedures are described in the materials and methods section.

**Replicates**

- You should report how often each experiment was performed
- You should include a definition of biological versus technical replication
- The data obtained should be provided and sufficient information should be provided to indicate the number of independent biological and/or technical replicates
- If you encountered any outliers, you should describe how these were handled
- Criteria for exclusion/inclusion of data should be clearly stated
- High-throughput sequence data should be uploaded before submission, with a private link for reviewers provided (these are available from both GEO and ArrayExpress)

Please outline where this information can be found within the submission (e.g., sections or figure legends), or explain why this information doesn’t apply to your submission:

• Phage and transduction titers were performed in triplicate as described in the materials and methods section and the average was reported. This is according to standard practices in the field.

• The calculated titers were used only to make an either/or decision of viability, which differed by at least four orders of magnitude.

• The interpretation of the data is described in Table 2 legend and in corresponding section in the results.

**Statistical reporting**

- Statistical analysis methods should be described and justified
- Raw data should be presented in figures whenever informative to do so (typically when N per group is less than 10)
- For each experiment, you should identify the statistical tests used, exact values of N, definitions of center, methods of multiple test correction, and dispersion and precision measures (e.g., mean, median, SD, SEM, confidence intervals; and, for the major substantive results, a measure of effect size (e.g., Pearson's r, Cohen's d)
- Report exact p-values wherever possible alongside the summary statistics and 95% confidence intervals. These should be reported for all key questions and not only when the p-value is less than 0.05.

Please outline where this information can be found within the submission (e.g., sections or figure legends), or explain why this information doesn’t apply to your submission:

• 3D reconstruction followed “gold standard” practices, in which the data was split into two half datasets, starting models were generated de novo from each subset, and FSC was calculated between the two half datasets.

• The molecular models were validated by refining against a half dataset (FSCwork) and comparing the resulting model against both FSCwork and an independent half (FSCfree).

• Reconstruction and modeling procedures are described in the materials and methods section.

• For effects of mutants on size determination, particles were counted and designated small procapsids, large procapsids, small virions and mature virions. In the results section, the % small capsids and total number of particles counted (n) are reported. One mutant which had a smaller percentage small capsids was subjected to a t-test against the other mutants with significance considered as p<0.05

(For large datasets, or papers with a very large number of statistical tests, you may upload a single table file with tests, Ns, etc., with reference to sections in the manuscript.)

**Group allocation**

- Indicate how samples were allocated into experimental groups (in the case of clinical studies, please specify allocation to treatment method); if randomization was used, please also state if restricted randomization was applied
- Indicate if masking was used during group allocation, data collection and/or data analysis

Please outline where this information can be found within the submission (e.g., sections or figure legends), or explain why this information doesn’t apply to your submission:

Not applicable to the present study.

**Additional data files (“source data”)**

- We encourage you to upload relevant additional data files, such as numerical data that are represented as a graph in a figure, or as a summary table
- Where provided, these should be in the most useful format, and they can be uploaded as “Source data” files linked to a main figure or table
- Include model definition files including the full list of parameters used
- Include code used for data analysis (e.g., R, MatLab)
- Avoid stating that data files are “available upon request”

Please indicate the figures or tables for which source data files have been provided:

The reconstructions and models have been submitted to EMDB and PDB with accession numbers EMDB-7030, PDB ID: 6B0X for 80alpha and EMDB-7035, PDB ID: 6B23 for SaPI1.
